# {α,α′-Bis[(*tert*-but­yl)(6-meth­oxy­pyridin-2-yl)phosphino]-*o*-xylene}(η^2^-*N*-methyl­maleinimide)palladium(0) toluene hemisolvate

**DOI:** 10.1107/S2414314625005085

**Published:** 2025-06-12

**Authors:** Stefan Müller, Anke Spannenberg, Helfried Neumann, Robert Franke, Matthias Beller

**Affiliations:** a Evonik Oxeno GmbH, Paul-Baumann-Str. 1, 45772 Marl, Germany; b Lehrstuhl für Theoretische Chemie, Ruhr-Universität Bochum, 44780 Bochum, Germany; c Leibniz-Institut für Katalyse e V Albert-Einstein-Str 29a 18059 Rostock Germany; University of Aberdeen, United Kingdom

**Keywords:** crystal structure, chelating ligand, palladium(0) complex, diphosphine ligand

## Abstract

The solvated title compound, [Pd(C_5_H_5_NO_2_)(C_28_H_38_N_2_O_2_P_2_)]·0.5C_7_H_8_, consists of a palladium(0) atom coordinated by a chelating α,α′-bis­[(*tert*-but­yl)(6-meth­oxy­pyridin-2-yl)phosphino]*o*-xylene ligand and an η^2^-coordinating *N*-methyl­maleinimide mol­ecule to generate a 16 electron complex in which the metal atom has a pseudo-square-planar coordination environment. The co-crystallized toluene solvent mol­ecule is disordered about an inversion centre by symmetry. Weak C—H⋯O and C—H⋯N hydrogen bonds connect the components in the extended structure.

## Abstract 2

The solvated title compound, [Pd(C_5_H_5_NO_2_)(C_28_H_38_N_2_O_2_P_2_)]·0.5C_7_H_8_, consists of a palladium(0) atom coordinated by a chelating α,α′-bis­[(*tert*-but­yl)(6-meth­oxy­pyridin-2-yl)phosphino]-*o*-xylene ligand and an η^2^-coordinating *N*-methyl­maleinimide molecule.

## Graphical abstract

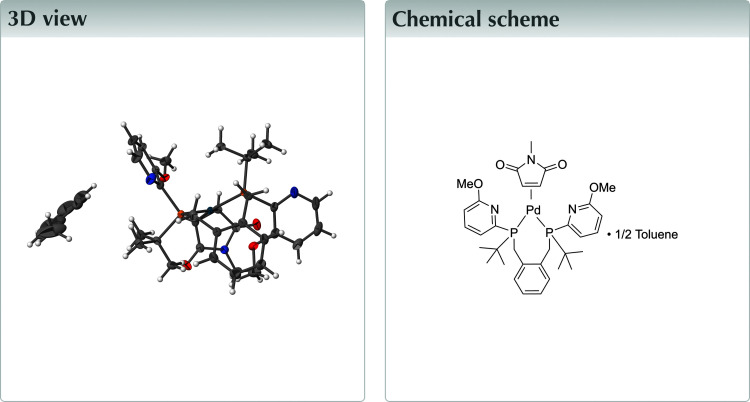

## Structure description

**Figure 1:**
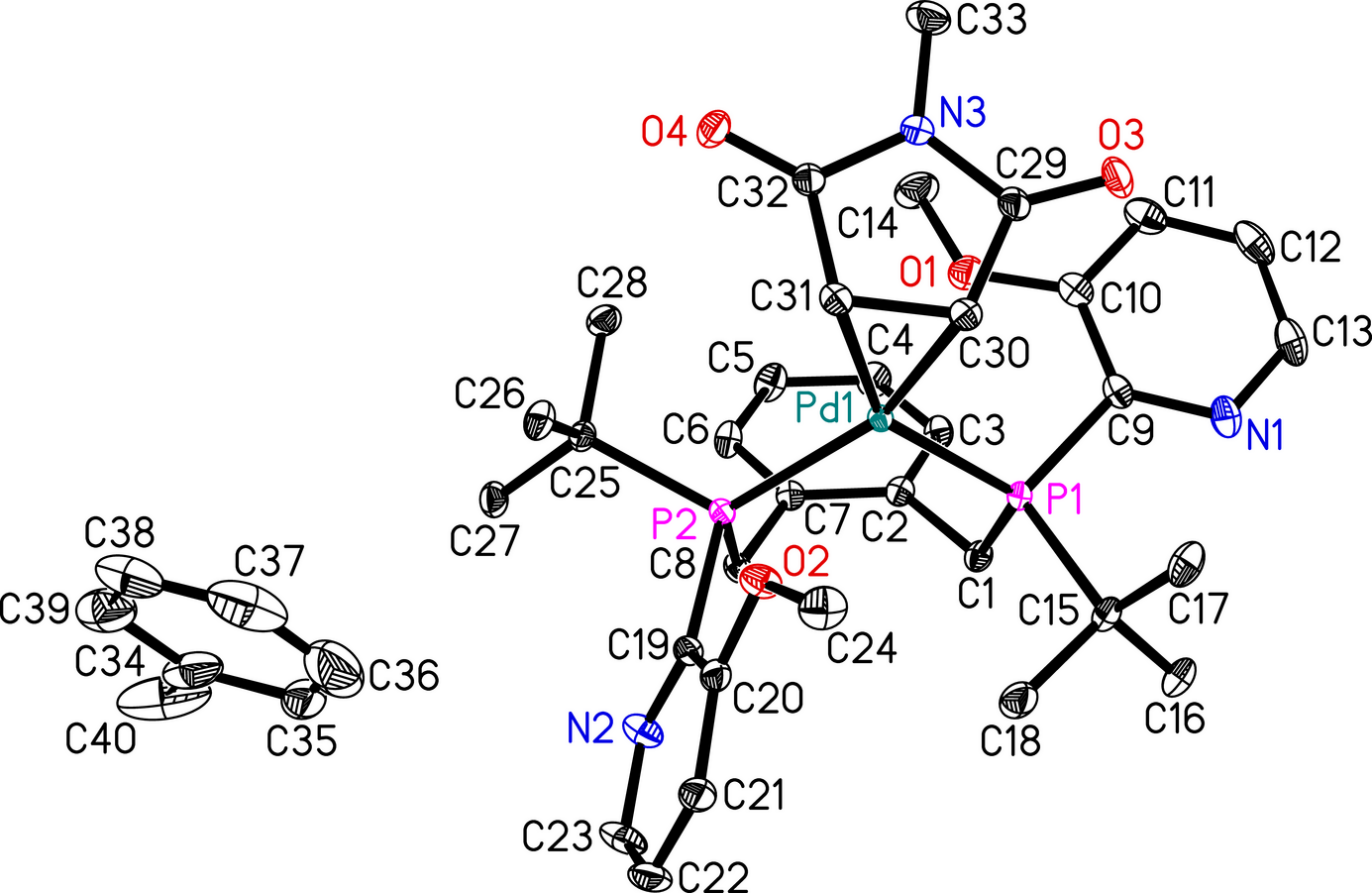
The mol­ecular structure of the title compound. Displacement ellipsoids correspond to 30% probability level. Hydrogen atoms are omitted for clarity. The toluene solvent molecule is shown with one of its possible orientations.

The parent ligand α,α′-bis­(di-*tert*-butyl­phosphino)-*o*-xylene shows a high activity in the palladium-catalysed meth­oxy­carbonyl­ation of olefins and is used in the Lucite Alpha process, where, on the hundred-thousand-tonne scale, ethyl­ene is meth­oxy­carbonyl­ated to methyl propionate (Eastham *et al.*, 2004 ▸). The substitution of one *tert*-butyl group by pyridyl gives α,α′-bis­[2-pyrid­yl(*tert*-but­yl)phosphino]-*o*-xylene, which shows a much higher activity in the palladium-catalysed meth­oxy­carbonyl­ation than the parent ligand (Dong *et al.*, 2017 ▸). Here, a palladium complex with α,α′-bis­[(*tert*-but­yl(6-meth­oxy­pyridin-2-yl)phosphino]-*o*-xylene, C_28_H_38_N_2_O_2_P_2_, is described, in which the metal is additionally η^2^-coordinated by the double bond of *N*-methyl­maleinimide, C_5_H_5_NO_2_. Thus, the central palladium atom has an oxidation number of 0 and exists as a 16-electron complex with pseudo-square-planar coordination environment. Since the phosphine ligand can exist as two diastereomers consisting of the *meso* and the racemic forms, the palladium complex can also occur in *meso* and racemic forms. The *meso* form has a mirror plane and the phospho­rous atoms are identical giving only one signal in the ^31^P NMR spectrum, while the racemic form is asymmetric. Consequently, the phospho­rous atoms in the latter form can couple with each other, showing two doublets in the ^31^P NMR spectrum. The racemic diastereomer is the major product of the ligand and the crystals arise in racemic form: in the arbitrarily chosen asymmetric mol­ecule (Fig. 1 ▸), both the P atoms have *R* configurations. The dihedral angles between the central C2–C7 ring and pendant N1/C9–C13 and N2/C19–C23 rings are 30.92 (10) and 68.65 (10)°, respectively. The C29–C32/N3 ring of the *N*-methyl­maleinimide ligand and the Pd1/C30/C31 grouping subtend a dihedral angle of 73.49 (14)°. Selected geometrical data are listed in Table 1 ▸. In the crystal, weak C—H⋯O and C—H⋯N hydrogen bonds (Table 2 ▸) link the mol­ecules.

## Synthesis and crystallization

17.98 mg (0.0845 mmol) of [(η^3^-all­yl)-η^5^-cyclo­penta­dien­yl]palladium complex was dissolved in 3 ml of absolute heptane and the red solution was filtrated over Celite into a 25 ml flask. A solution made of 42 mg (0.0845 mmol) of the ligand α,α′-bis­[(*tert*-but­yl)(6-meth­oxy­pyridin-2-yl)phosphino]-*o*-xylene and 9.29 mg (0.0845 mmol) *N*-methyl­maleinimide in 7 ml of heptane were added slowly to the deep-red filtrate. The reaction solution was decolourized to pale pink, and a bright-yellow precipitate was formed. After 2 days the reaction solution was deca­ntated and the white solid was washed three times with 5 ml heptane each. After drying in a vacuum, 42 mg (70%) of a white solid was obtained. A phospho­rous NMR of the complex was recorded using toluene-*d*_8_ as a solvent.

^31^P NMR (121 Hz, toluene-*d*_8_) δ 26.10 (*s*), 23.71 (*d*, *J* = 27.7 Hz), 23.04 (*d*, *J* = 27.6 Hz).

The singlet signal is associated with the *meso* form of the palladium complex, while the two doublets belong to the racemic title complex. Since the NMR solution was slightly cloudy, 0.8 ml of toluene-*d*_8_ were added and the solution was filtrated over Celite. The clear solution was transferred under argon into a 4 ml vial, which was located in a bigger Schlenk flask filled with glass sticks. After closing by a septum, 10 ml of pentane were added *via* syringe into the larger Schlenk flask and the septum was replaced by a stopper. After 3 days, tiny crystals of the title compound, suitable for X-ray analysis, were formed by diffusion of pentane into the toluene phase.

## Refinement

Crystal data, data collection and structure refinement details are summarized in Table 3 ▸. H30 and H31 could be found from the difference-Fourier map and were refined freely. AFIX 66 and *DFIX* commands in *SHELXL* were used to optimize the geometry of the half-occupied toluene mol­ecule and the SIMU instruction was included to equalize the displacement parameters of their non-hydrogen atoms (C34–C40).

## Supplementary Material

Crystal structure: contains datablock(s) I. DOI: 10.1107/S2414314625005085/hb4516sup1.cif

Structure factors: contains datablock(s) I. DOI: 10.1107/S2414314625005085/hb4516Isup2.hkl

CCDC reference: 2456579

Additional supporting information:  crystallographic information; 3D view; checkCIF report

## Figures and Tables

**Table 1:** Selected geometric parameters (Å, °)

Pd1—C30	2.1174 (17)	Pd1—P1	2.2965 (4)
Pd1—C31	2.1343 (17)	Pd1—P2	2.3058 (4)
			
C30—Pd1—C31	39.36 (7)	C30—Pd1—P2	149.41 (5)
C30—Pd1—P1	107.67 (5)	C31—Pd1—P2	111.69 (5)
C31—Pd1—P1	146.86 (5)	P1—Pd1—P2	101.314 (15)

**Table 2:** Hydrogen-bond geometry (Å, °)

*D*—H⋯*A*	*D*—H	H⋯*A*	*D*⋯*A*	*D*—H⋯*A*
C3—H3⋯O4^i^	0.95	2.54	3.480 (2)	172
C12—H12⋯O3^ii^	0.95	2.37	3.281 (3)	161
C38—H38⋯N2^iii^	0.95	2.60	3.462 (5)	151

Symmetry codes: (i) 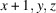; (ii) 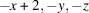; (iii) 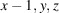.

**Table 3:** Experimental details

Crystal data	
Chemical formula	[Pd(C_5_H_5_NO_2_)(C_28_H_38_N_2_O_2_P_2_)]·0.5C_7_H_8_
*M*_r_	760.11
Crystal system, space group	Triclinic, *P* 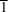
Temperature (K)	150
*a*, *b*, *c* (Å)	9.5548 (2), 10.7020 (3), 18.4654 (5)
α, β, γ (°)	74.0508 (10), 86.9998 (10), 76.0888 (9)
*V* (Å^3^)	1762.06 (8)
*Z*	2
Radiation type	Cu *K*α
μ (mm^−1^)	5.45
Crystal size (mm)	0.19 × 0.08 × 0.03

Data collection	
Diffractometer	Bruker APEXII CCD
Absorption correction	Multi-scan (*SADABS*; Krause *et al.*, 2015 ▸)
*T*_min_, *T*_max_	0.42, 0.85
No. of measured, independent and observed [*I* > 2σ(*I*)] reflections	27460, 6223, 5975
*R*_int_	0.030
(sin θ/λ)_max_ (Å^−1^)	0.596

Refinement	
*R*[*F*^2^ > 2σ(*F*^2^)], *wR*(*F*^2^), *S*	0.022, 0.057, 1.06
No. of reflections	6223
No. of parameters	456
No. of restraints	43
H-atom treatment	H atoms treated by a mixture of independent and constrained refinement
Δρ_max_, Δρ_min_ (e Å^−3^)	0.38, −0.32

Computer programs: *APEX2* (Bruker, 2014 ▸), *SAINT* (Bruker, 2013 ▸), *SHELXT* (Sheldrick, 2015*a* ▸), *SHELXL2019/1* (Sheldrick, 2015*b* ▸), *XP* in *SHELXTL* (Sheldrick, 2008 ▸) and *publCIF* (Westrip, 2010 ▸).

## full crystallographic data

### (α,α'-Bis[(tert-butyl)(6-methoxypyridin-2-yl)phosphino]-o-xylene}(η2-N-methylmaleinimide)palladium(0) toluene hemisolvate Crystal data

**Table:**

[Pd(C_5_H_5_NO_2_)(C_28_H_38_N_2_O_2_P_2_)]·0.5C_7_H_8_	*Z* = 2
*M**_r_* = 760.11	*F*(000) = 790
Triclinic, *P*1	*D*_x_ = 1.433 Mg m^−^^3^
*a* = 9.5548 (2) Å	Cu *K*α radiation, λ = 1.54178 Å
*b* = 10.7020 (3) Å	Cell parameters from 9834 reflections
*c* = 18.4654 (5) Å	θ = 2.5–66.6°
α = 74.0508 (10)°	µ = 5.45 mm^−^^1^
β = 86.9998 (10)°	*T* = 150 K
γ = 76.0888 (9)°	Plate, colourless
*V* = 1762.06 (8) Å^3^	0.19 × 0.08 × 0.03 mm

### (α,α'-Bis[(tert-butyl)(6-methoxypyridin-2-yl)phosphino]-o-xylene}(η2-N-methylmaleinimide)palladium(0) toluene hemisolvate Data collection

**Table:**

Bruker APEXII CCD diffractometer	6223 independent reflections
Radiation source: microfocus	5975 reflections with *I* > 2σ(*I*)
Multilayer monochromator	*R*_int_ = 0.030
φ and ω scans	θ_max_ = 66.7°, θ_min_ = 2.5°
Absorption correction: multi-scan (SADABS; Krause *et al.*, 2015)	*h* = −10→11
*T*_min_ = 0.42, *T*_max_ = 0.85	*k* = −12→12
27460 measured reflections	*l* = −21→21

### (α,α'-Bis[(tert-butyl)(6-methoxypyridin-2-yl)phosphino]-o-xylene}(η2-N-methylmaleinimide)palladium(0) toluene hemisolvate Refinement

**Table:**

Refinement on *F*^2^	Primary atom site location: dual
Least-squares matrix: full	Hydrogen site location: mixed
*R*[*F*^2^ > 2σ(*F*^2^)] = 0.022	H atoms treated by a mixture of independent and constrained refinement
*wR*(*F*^2^) = 0.057	*w* = 1/[σ^2^(*F*_o_^2^) + (0.0327*P*)^2^ + 0.6517*P*] where *P* = (*F*_o_^2^ + 2*F*_c_^2^)/3
*S* = 1.06	(Δ/σ)_max_ = 0.001
6223 reflections	Δρ_max_ = 0.38 e Å^−^^3^
456 parameters	Δρ_min_ = −0.32 e Å^−^^3^
43 restraints	

### (α,α'-Bis[(tert-butyl)(6-methoxypyridin-2-yl)phosphino]-o-xylene}(η2-N-methylmaleinimide)palladium(0) toluene hemisolvate Special details

**Table:**

Geometry. All esds (except the esd in the dihedral angle between two l.s. planes) are estimated using the full covariance matrix. The cell esds are taken into account individually in the estimation of esds in distances, angles and torsion angles; correlations between esds in cell parameters are only used when they are defined by crystal symmetry. An approximate (isotropic) treatment of cell esds is used for estimating esds involving l.s. planes.

### (α,α'-Bis[(tert-butyl)(6-methoxypyridin-2-yl)phosphino]-o-xylene}(η2-N-methylmaleinimide)palladium(0) toluene hemisolvate Fractional atomic coordinates and isotropic or equivalent isotropic displacement parameters (Å^2^)

**Table:**

	*x*	*y*	*z*	*U*_iso_*/*U*_eq_	Occ. (<1)
C1	0.93696 (19)	0.27389 (19)	0.26501 (10)	0.0219 (4)	
H1A	1.038026	0.265442	0.248101	0.026*	
H1B	0.911415	0.351279	0.286668	0.026*	
C2	0.92856 (19)	0.14923 (19)	0.32643 (10)	0.0208 (4)	
C3	1.0378 (2)	0.0339 (2)	0.33169 (11)	0.0264 (4)	
H3	1.112812	0.034881	0.295842	0.032*	
C4	1.0385 (2)	−0.0822 (2)	0.38842 (13)	0.0317 (4)	
H4	1.112981	−0.160168	0.390936	0.038*	
C5	0.9305 (2)	−0.0842 (2)	0.44141 (12)	0.0329 (5)	
H5	0.930161	−0.163591	0.480292	0.039*	
C6	0.8230 (2)	0.0302 (2)	0.43723 (11)	0.0271 (4)	
H6	0.750048	0.028730	0.474249	0.033*	
C7	0.81861 (19)	0.14829 (19)	0.37994 (10)	0.0209 (4)	
C8	0.70393 (19)	0.27224 (19)	0.38093 (10)	0.0217 (4)	
H8A	0.748903	0.349664	0.366498	0.026*	
H8B	0.670427	0.263624	0.433334	0.026*	
C9	0.9288 (2)	0.18911 (19)	0.13448 (10)	0.0227 (4)	
C10	0.9108 (2)	0.0574 (2)	0.15543 (11)	0.0271 (4)	
C11	0.9976 (2)	−0.0365 (2)	0.12297 (13)	0.0359 (5)	
H11	0.988207	−0.126339	0.136391	0.043*	
C12	1.0977 (2)	0.0040 (3)	0.07083 (13)	0.0403 (5)	
H12	1.158779	−0.057616	0.047661	0.048*	
C13	1.1076 (2)	0.1352 (3)	0.05293 (13)	0.0397 (5)	
H13	1.176471	0.162079	0.016699	0.048*	
C14	0.8098 (3)	−0.1042 (2)	0.24477 (16)	0.0447 (6)	
H14A	0.788263	−0.149860	0.208870	0.067*	
H14B	0.737396	−0.107416	0.284441	0.067*	
H14C	0.905612	−0.148857	0.267321	0.067*	
C15	0.8384 (2)	0.47990 (18)	0.12717 (11)	0.0239 (4)	
C16	0.9905 (2)	0.5028 (2)	0.12827 (12)	0.0314 (4)	
H16A	1.058980	0.435590	0.109059	0.047*	
H16B	1.017764	0.495027	0.180035	0.047*	
H16C	0.992061	0.592304	0.096426	0.047*	
C17	0.7880 (2)	0.5048 (2)	0.04594 (12)	0.0339 (5)	
H17A	0.790373	0.596066	0.017312	0.051*	
H17B	0.689335	0.493195	0.045913	0.051*	
H17C	0.852119	0.440888	0.022626	0.051*	
C18	0.7345 (2)	0.5786 (2)	0.16348 (14)	0.0365 (5)	
H18A	0.737224	0.670312	0.135890	0.055*	
H18B	0.763806	0.561504	0.216044	0.055*	
H18C	0.636309	0.566724	0.161636	0.055*	
C19	0.47054 (19)	0.48686 (18)	0.32103 (11)	0.0217 (4)	
C20	0.3886 (2)	0.58387 (18)	0.26075 (11)	0.0233 (4)	
C21	0.3500 (2)	0.7168 (2)	0.26300 (13)	0.0299 (4)	
H21	0.297344	0.784371	0.222513	0.036*	
C22	0.3900 (2)	0.7482 (2)	0.32514 (14)	0.0373 (5)	
H22	0.366071	0.838214	0.327934	0.045*	
C23	0.4650 (3)	0.6476 (2)	0.38326 (15)	0.0406 (5)	
H23	0.488761	0.670010	0.426669	0.049*	
C24	0.3017 (2)	0.6378 (2)	0.13411 (12)	0.0342 (5)	
H24A	0.207408	0.693147	0.141900	0.051*	
H24B	0.292520	0.592165	0.095883	0.051*	
H24C	0.370151	0.694553	0.117083	0.051*	
C25	0.4107 (2)	0.22225 (18)	0.37693 (10)	0.0211 (4)	
C26	0.2622 (2)	0.2889 (2)	0.33945 (11)	0.0278 (4)	
H26A	0.190948	0.241256	0.366123	0.042*	
H26B	0.266164	0.285940	0.286793	0.042*	
H26C	0.234534	0.382210	0.341447	0.042*	
C27	0.4047 (2)	0.2302 (2)	0.45863 (11)	0.0276 (4)	
H27A	0.389840	0.323658	0.459359	0.041*	
H27B	0.495543	0.177977	0.484686	0.041*	
H27C	0.324687	0.193929	0.484133	0.041*	
C28	0.4531 (2)	0.07631 (19)	0.37335 (11)	0.0283 (4)	
H28A	0.549613	0.033947	0.395548	0.042*	
H28B	0.453223	0.073754	0.320696	0.042*	
H28C	0.383498	0.028081	0.401506	0.042*	
C29	0.5627 (2)	0.1729 (2)	0.07110 (10)	0.0238 (4)	
C30	0.5027 (2)	0.29784 (19)	0.09226 (10)	0.0207 (4)	
C31	0.39618 (19)	0.27265 (19)	0.14840 (10)	0.0208 (4)	
C32	0.3922 (2)	0.13104 (19)	0.16241 (10)	0.0229 (4)	
C33	0.5262 (3)	−0.0605 (2)	0.11249 (14)	0.0378 (5)	
H33A	0.450360	−0.073669	0.083619	0.057*	
H33B	0.530729	−0.119478	0.163797	0.057*	
H33C	0.619208	−0.081624	0.088129	0.057*	
N1	1.02564 (19)	0.22701 (19)	0.08374 (10)	0.0318 (4)	
N2	0.5060 (2)	0.51956 (18)	0.38144 (10)	0.0316 (4)	
N3	0.49423 (18)	0.07741 (16)	0.11534 (9)	0.0249 (3)	
O1	0.80689 (16)	0.03170 (14)	0.20648 (9)	0.0319 (3)	
O2	0.35302 (15)	0.54032 (13)	0.20356 (8)	0.0267 (3)	
O3	0.65229 (16)	0.15087 (16)	0.02391 (8)	0.0345 (3)	
O4	0.31817 (16)	0.06561 (16)	0.20498 (8)	0.0327 (3)	
P1	0.81919 (4)	0.30985 (4)	0.18162 (2)	0.01687 (9)	
P2	0.54364 (4)	0.31042 (4)	0.31899 (2)	0.01620 (9)	
Pd1	0.58477 (2)	0.29018 (2)	0.19811 (2)	0.01527 (5)	
H30	0.500 (3)	0.380 (3)	0.0561 (15)	0.040 (7)*	
H31	0.316 (3)	0.330 (3)	0.1550 (14)	0.033 (6)*	
C34	0.0369 (5)	0.4754 (5)	0.5408 (3)	0.0581 (15)	0.5
C35	0.1062 (5)	0.5331 (4)	0.4767 (4)	0.060 (3)	0.5
H35	0.200799	0.544515	0.480700	0.072*	0.5
C36	0.0370 (7)	0.5742 (5)	0.4069 (3)	0.089 (2)	0.5
H36	0.084303	0.613630	0.363106	0.107*	0.5
C37	−0.1015 (7)	0.5575 (5)	0.4011 (3)	0.089 (3)	0.5
H37	−0.148770	0.585542	0.353366	0.106*	0.5
C38	−0.1707 (5)	0.4997 (5)	0.4652 (3)	0.0645 (16)	0.5
H38	−0.265348	0.488339	0.461220	0.077*	0.5
C39	−0.1015 (5)	0.4587 (4)	0.5350 (3)	0.061 (3)	0.5
H39	−0.148854	0.419223	0.578814	0.074*	0.5
C40	0.1089 (12)	0.4269 (10)	0.6172 (6)	0.103 (4)	0.5
H40A	0.042980	0.389700	0.654788	0.155*	0.5
H40B	0.196761	0.357626	0.616414	0.155*	0.5
H40C	0.133824	0.501869	0.630119	0.155*	0.5

### (α,α'-Bis[(tert-butyl)(6-methoxypyridin-2-yl)phosphino]-o-xylene}(η2-N-methylmaleinimide)palladium(0) toluene hemisolvate Atomic displacement parameters (Å^2^)

**Table:**

	*U*^11^	*U*^22^	*U*^33^	*U*^12^	*U*^13^	*U*^23^
C1	0.0191 (9)	0.0263 (9)	0.0203 (9)	−0.0080 (7)	−0.0011 (7)	−0.0037 (7)
C2	0.0181 (9)	0.0250 (9)	0.0190 (9)	−0.0058 (7)	−0.0041 (7)	−0.0038 (7)
C3	0.0178 (9)	0.0324 (10)	0.0274 (10)	−0.0033 (7)	0.0005 (7)	−0.0078 (8)
C4	0.0232 (10)	0.0272 (10)	0.0382 (11)	0.0018 (8)	−0.0024 (8)	−0.0044 (9)
C5	0.0288 (11)	0.0294 (10)	0.0308 (11)	−0.0038 (8)	−0.0027 (8)	0.0056 (8)
C6	0.0206 (9)	0.0344 (10)	0.0220 (9)	−0.0055 (8)	0.0005 (7)	−0.0014 (8)
C7	0.0177 (9)	0.0261 (9)	0.0193 (9)	−0.0053 (7)	−0.0032 (6)	−0.0062 (7)
C8	0.0197 (9)	0.0274 (9)	0.0191 (9)	−0.0043 (7)	−0.0004 (7)	−0.0092 (7)
C9	0.0190 (9)	0.0262 (9)	0.0202 (9)	0.0012 (7)	−0.0015 (7)	−0.0073 (7)
C10	0.0246 (10)	0.0284 (10)	0.0268 (10)	0.0001 (8)	−0.0044 (7)	−0.0097 (8)
C11	0.0337 (11)	0.0308 (11)	0.0410 (12)	0.0052 (9)	−0.0069 (9)	−0.0158 (9)
C12	0.0326 (12)	0.0479 (14)	0.0365 (12)	0.0119 (10)	−0.0020 (9)	−0.0231 (10)
C13	0.0295 (11)	0.0528 (14)	0.0324 (11)	0.0020 (10)	0.0090 (9)	−0.0160 (10)
C14	0.0527 (15)	0.0245 (11)	0.0551 (15)	−0.0122 (10)	0.0019 (11)	−0.0055 (10)
C15	0.0202 (9)	0.0211 (9)	0.0272 (10)	−0.0064 (7)	0.0038 (7)	−0.0008 (7)
C16	0.0257 (10)	0.0325 (11)	0.0341 (11)	−0.0142 (8)	0.0024 (8)	0.0000 (8)
C17	0.0323 (11)	0.0350 (11)	0.0278 (11)	−0.0122 (9)	−0.0026 (8)	0.0067 (8)
C18	0.0367 (12)	0.0205 (10)	0.0486 (13)	−0.0057 (8)	0.0117 (10)	−0.0062 (9)
C19	0.0191 (9)	0.0207 (9)	0.0266 (9)	−0.0040 (7)	0.0042 (7)	−0.0099 (7)
C20	0.0212 (9)	0.0209 (9)	0.0278 (10)	−0.0053 (7)	0.0056 (7)	−0.0071 (7)
C21	0.0288 (10)	0.0205 (9)	0.0395 (12)	−0.0036 (8)	0.0067 (8)	−0.0096 (8)
C22	0.0375 (12)	0.0239 (10)	0.0564 (14)	−0.0057 (9)	0.0075 (10)	−0.0232 (10)
C23	0.0432 (13)	0.0380 (12)	0.0502 (14)	−0.0044 (10)	−0.0023 (10)	−0.0317 (11)
C24	0.0393 (12)	0.0258 (10)	0.0308 (11)	−0.0025 (8)	−0.0102 (9)	0.0006 (8)
C25	0.0216 (9)	0.0240 (9)	0.0187 (9)	−0.0087 (7)	0.0029 (7)	−0.0047 (7)
C26	0.0204 (9)	0.0356 (11)	0.0272 (10)	−0.0105 (8)	0.0023 (7)	−0.0053 (8)
C27	0.0294 (10)	0.0348 (10)	0.0201 (9)	−0.0112 (8)	0.0056 (7)	−0.0074 (8)
C28	0.0349 (11)	0.0240 (10)	0.0270 (10)	−0.0121 (8)	0.0017 (8)	−0.0043 (8)
C29	0.0238 (9)	0.0329 (10)	0.0180 (9)	−0.0094 (8)	−0.0015 (7)	−0.0098 (7)
C30	0.0230 (9)	0.0239 (9)	0.0155 (8)	−0.0068 (7)	−0.0047 (7)	−0.0037 (7)
C31	0.0159 (9)	0.0262 (9)	0.0212 (9)	−0.0026 (7)	−0.0032 (7)	−0.0092 (7)
C32	0.0207 (9)	0.0306 (10)	0.0206 (9)	−0.0088 (7)	−0.0014 (7)	−0.0096 (7)
C33	0.0478 (14)	0.0267 (11)	0.0427 (13)	−0.0076 (9)	0.0027 (10)	−0.0172 (9)
N1	0.0263 (9)	0.0379 (10)	0.0278 (9)	−0.0016 (7)	0.0059 (7)	−0.0093 (7)
N2	0.0337 (9)	0.0321 (9)	0.0324 (9)	−0.0027 (7)	−0.0032 (7)	−0.0182 (7)
N3	0.0278 (8)	0.0246 (8)	0.0264 (8)	−0.0092 (6)	0.0039 (6)	−0.0115 (7)
O1	0.0336 (8)	0.0201 (7)	0.0396 (8)	−0.0038 (6)	0.0051 (6)	−0.0071 (6)
O2	0.0333 (7)	0.0175 (6)	0.0255 (7)	0.0008 (5)	−0.0056 (5)	−0.0045 (5)
O3	0.0344 (8)	0.0497 (9)	0.0267 (7)	−0.0140 (7)	0.0114 (6)	−0.0206 (7)
O4	0.0329 (8)	0.0432 (8)	0.0292 (7)	−0.0227 (7)	0.0070 (6)	−0.0110 (6)
P1	0.0154 (2)	0.0181 (2)	0.0162 (2)	−0.00396 (15)	0.00148 (15)	−0.00328 (16)
P2	0.0158 (2)	0.0173 (2)	0.0159 (2)	−0.00310 (15)	0.00078 (15)	−0.00596 (16)
Pd1	0.01458 (7)	0.01625 (7)	0.01513 (7)	−0.00324 (5)	0.00050 (4)	−0.00487 (5)
C34	0.044 (3)	0.030 (2)	0.102 (4)	−0.007 (2)	−0.013 (3)	−0.020 (3)
C35	0.051 (5)	0.035 (4)	0.101 (5)	−0.015 (4)	0.030 (4)	−0.029 (4)
C36	0.122 (5)	0.047 (3)	0.092 (5)	−0.008 (4)	0.039 (5)	−0.026 (3)
C37	0.129 (7)	0.053 (5)	0.087 (5)	−0.003 (5)	−0.014 (5)	−0.038 (4)
C38	0.077 (4)	0.045 (3)	0.078 (4)	−0.002 (3)	−0.014 (3)	−0.036 (3)
C39	0.042 (4)	0.042 (4)	0.103 (5)	−0.001 (4)	−0.013 (4)	−0.029 (4)
C40	0.118 (8)	0.045 (4)	0.144 (8)	−0.016 (5)	−0.085 (7)	−0.007 (5)

### (α,α'-Bis[(tert-butyl)(6-methoxypyridin-2-yl)phosphino]-o-xylene}(η2-N-methylmaleinimide)palladium(0) toluene hemisolvate Geometric parameters (Å, º)

**Table:**

C1—C2	1.511 (2)	C22—C23	1.378 (4)
C1—P1	1.8528 (18)	C22—H22	0.9500
C1—H1A	0.9900	C23—N2	1.341 (3)
C1—H1B	0.9900	C23—H23	0.9500
C2—C3	1.396 (3)	C24—O2	1.434 (2)
C2—C7	1.404 (3)	C24—H24A	0.9800
C3—C4	1.386 (3)	C24—H24B	0.9800
C3—H3	0.9500	C24—H24C	0.9800
C4—C5	1.384 (3)	C25—C27	1.531 (3)
C4—H4	0.9500	C25—C26	1.532 (3)
C5—C6	1.383 (3)	C25—C28	1.535 (3)
C5—H5	0.9500	C25—P2	1.8779 (18)
C6—C7	1.401 (3)	C26—H26A	0.9800
C6—H6	0.9500	C26—H26B	0.9800
C7—C8	1.508 (2)	C26—H26C	0.9800
C8—P2	1.8564 (18)	C27—H27A	0.9800
C8—H8A	0.9900	C27—H27B	0.9800
C8—H8B	0.9900	C27—H27C	0.9800
C9—N1	1.334 (3)	C28—H28A	0.9800
C9—C10	1.405 (3)	C28—H28B	0.9800
C9—P1	1.8427 (19)	C28—H28C	0.9800
C10—O1	1.357 (3)	C29—O3	1.219 (2)
C10—C11	1.391 (3)	C29—N3	1.395 (2)
C11—C12	1.379 (4)	C29—C30	1.471 (3)
C11—H11	0.9500	C30—C31	1.432 (3)
C12—C13	1.376 (4)	Pd1—C30	2.1174 (17)
C12—H12	0.9500	Pd1—C31	2.1343 (17)
C13—N1	1.339 (3)	C30—H30	0.95 (3)
C13—H13	0.9500	C31—C32	1.475 (3)
C14—O1	1.427 (3)	C31—H31	0.88 (3)
C14—H14A	0.9800	C32—O4	1.219 (2)
C14—H14B	0.9800	C32—N3	1.397 (2)
C14—H14C	0.9800	C33—N3	1.449 (3)
C15—C17	1.532 (3)	C33—H33A	0.9800
C15—C16	1.533 (3)	C33—H33B	0.9800
C15—C18	1.538 (3)	C33—H33C	0.9800
C15—P1	1.8687 (18)	Pd1—P1	2.2965 (4)
C16—H16A	0.9800	Pd1—P2	2.3058 (4)
C16—H16B	0.9800	C34—C35	1.3900
C16—H16C	0.9800	C34—C39	1.3900
C17—H17A	0.9800	C34—C40	1.503 (8)
C17—H17B	0.9800	C35—C36	1.3900
C17—H17C	0.9800	C35—H35	0.9500
C18—H18A	0.9800	C36—C37	1.3900
C18—H18B	0.9800	C36—H36	0.9500
C18—H18C	0.9800	C37—C38	1.3900
C19—N2	1.339 (3)	C37—H37	0.9500
C19—C20	1.413 (3)	C38—C39	1.3900
C19—P2	1.8593 (18)	C38—H38	0.9500
C20—O2	1.353 (2)	C39—H39	0.9500
C20—C21	1.393 (3)	C40—H40A	0.9800
C21—C22	1.376 (3)	C40—H40B	0.9800
C21—H21	0.9500	C40—H40C	0.9800
			
C2—C1—P1	115.20 (13)	C27—C25—C28	111.00 (16)
C2—C1—H1A	108.5	C26—C25—C28	109.01 (16)
P1—C1—H1A	108.5	C27—C25—P2	113.04 (13)
C2—C1—H1B	108.5	C26—C25—P2	107.15 (12)
P1—C1—H1B	108.5	C28—C25—P2	107.33 (12)
H1A—C1—H1B	107.5	C25—C26—H26A	109.5
C3—C2—C7	119.52 (17)	C25—C26—H26B	109.5
C3—C2—C1	118.43 (17)	H26A—C26—H26B	109.5
C7—C2—C1	121.98 (17)	C25—C26—H26C	109.5
C4—C3—C2	121.02 (18)	H26A—C26—H26C	109.5
C4—C3—H3	119.5	H26B—C26—H26C	109.5
C2—C3—H3	119.5	C25—C27—H27A	109.5
C5—C4—C3	119.93 (19)	C25—C27—H27B	109.5
C5—C4—H4	120.0	H27A—C27—H27B	109.5
C3—C4—H4	120.0	C25—C27—H27C	109.5
C6—C5—C4	119.41 (19)	H27A—C27—H27C	109.5
C6—C5—H5	120.3	H27B—C27—H27C	109.5
C4—C5—H5	120.3	C25—C28—H28A	109.5
C5—C6—C7	121.84 (18)	C25—C28—H28B	109.5
C5—C6—H6	119.1	H28A—C28—H28B	109.5
C7—C6—H6	119.1	C25—C28—H28C	109.5
C6—C7—C2	118.26 (17)	H28A—C28—H28C	109.5
C6—C7—C8	119.43 (17)	H28B—C28—H28C	109.5
C2—C7—C8	122.14 (16)	O3—C29—N3	123.79 (19)
C7—C8—P2	116.59 (13)	O3—C29—C30	129.50 (18)
C7—C8—H8A	108.1	N3—C29—C30	106.70 (16)
P2—C8—H8A	108.1	C31—C30—C29	107.62 (16)
C7—C8—H8B	108.1	C31—C30—Pd1	70.96 (10)
P2—C8—H8B	108.1	C29—C30—Pd1	111.37 (12)
H8A—C8—H8B	107.3	C31—C30—H30	125.4 (16)
N1—C9—C10	121.87 (18)	C29—C30—H30	119.0 (16)
N1—C9—P1	119.68 (15)	Pd1—C30—H30	112.6 (16)
C10—C9—P1	118.41 (14)	C30—C31—C32	107.13 (16)
O1—C10—C11	124.5 (2)	C30—C31—Pd1	69.68 (10)
O1—C10—C9	116.40 (17)	C32—C31—Pd1	110.84 (12)
C11—C10—C9	119.1 (2)	C30—C31—H31	126.7 (16)
C12—C11—C10	118.4 (2)	C32—C31—H31	118.1 (16)
C12—C11—H11	120.8	Pd1—C31—H31	114.6 (16)
C10—C11—H11	120.8	O4—C32—N3	122.77 (18)
C13—C12—C11	118.9 (2)	O4—C32—C31	130.48 (18)
C13—C12—H12	120.5	N3—C32—C31	106.75 (16)
C11—C12—H12	120.5	N3—C33—H33A	109.5
N1—C13—C12	123.7 (2)	N3—C33—H33B	109.5
N1—C13—H13	118.2	H33A—C33—H33B	109.5
C12—C13—H13	118.2	N3—C33—H33C	109.5
O1—C14—H14A	109.5	H33A—C33—H33C	109.5
O1—C14—H14B	109.5	H33B—C33—H33C	109.5
H14A—C14—H14B	109.5	C9—N1—C13	118.1 (2)
O1—C14—H14C	109.5	C19—N2—C23	118.76 (19)
H14A—C14—H14C	109.5	C29—N3—C32	111.78 (16)
H14B—C14—H14C	109.5	C29—N3—C33	123.69 (17)
C17—C15—C16	110.28 (16)	C32—N3—C33	124.53 (17)
C17—C15—C18	108.77 (17)	C10—O1—C14	118.36 (17)
C16—C15—C18	108.78 (17)	C20—O2—C24	118.15 (15)
C17—C15—P1	108.17 (14)	C9—P1—C1	98.98 (8)
C16—C15—P1	114.86 (13)	C9—P1—C15	107.32 (9)
C18—C15—P1	105.75 (13)	C1—P1—C15	102.25 (8)
C15—C16—H16A	109.5	C9—P1—Pd1	112.72 (6)
C15—C16—H16B	109.5	C1—P1—Pd1	119.64 (6)
H16A—C16—H16B	109.5	C15—P1—Pd1	114.18 (6)
C15—C16—H16C	109.5	C8—P2—C19	98.06 (8)
H16A—C16—H16C	109.5	C8—P2—C25	106.08 (8)
H16B—C16—H16C	109.5	C19—P2—C25	102.72 (8)
C15—C17—H17A	109.5	C8—P2—Pd1	117.16 (6)
C15—C17—H17B	109.5	C19—P2—Pd1	112.36 (6)
H17A—C17—H17B	109.5	C25—P2—Pd1	117.81 (6)
C15—C17—H17C	109.5	C30—Pd1—C31	39.36 (7)
H17A—C17—H17C	109.5	C30—Pd1—P1	107.67 (5)
H17B—C17—H17C	109.5	C31—Pd1—P1	146.86 (5)
C15—C18—H18A	109.5	C30—Pd1—P2	149.41 (5)
C15—C18—H18B	109.5	C31—Pd1—P2	111.69 (5)
H18A—C18—H18B	109.5	P1—Pd1—P2	101.314 (15)
C15—C18—H18C	109.5	C35—C34—C39	120.0
H18A—C18—H18C	109.5	C35—C34—C40	121.8 (6)
H18B—C18—H18C	109.5	C39—C34—C40	118.2 (6)
N2—C19—C20	121.18 (17)	C34—C35—C36	120.0
N2—C19—P2	116.31 (14)	C34—C35—H35	120.0
C20—C19—P2	122.42 (14)	C36—C35—H35	120.0
O2—C20—C21	124.05 (18)	C35—C36—C37	120.0
O2—C20—C19	116.89 (16)	C35—C36—H36	120.0
C21—C20—C19	119.06 (18)	C37—C36—H36	120.0
C22—C21—C20	118.6 (2)	C38—C37—C36	120.0
C22—C21—H21	120.7	C38—C37—H37	120.0
C20—C21—H21	120.7	C36—C37—H37	120.0
C21—C22—C23	119.22 (19)	C39—C38—C37	120.0
C21—C22—H22	120.4	C39—C38—H38	120.0
C23—C22—H22	120.4	C37—C38—H38	120.0
N2—C23—C22	123.1 (2)	C38—C39—C34	120.0
N2—C23—H23	118.4	C38—C39—H39	120.0
C22—C23—H23	118.4	C34—C39—H39	120.0
O2—C24—H24A	109.5	C34—C40—H40A	109.5
O2—C24—H24B	109.5	C34—C40—H40B	109.5
H24A—C24—H24B	109.5	H40A—C40—H40B	109.5
O2—C24—H24C	109.5	C34—C40—H40C	109.5
H24A—C24—H24C	109.5	H40A—C40—H40C	109.5
H24B—C24—H24C	109.5	H40B—C40—H40C	109.5
C27—C25—C26	109.17 (16)		

### (α,α'-Bis[(tert-butyl)(6-methoxypyridin-2-yl)phosphino]-o-xylene}(η2-N-methylmaleinimide)palladium(0) toluene hemisolvate Hydrogen-bond geometry (Å, º)

**Table:**

*D*—H···*A*	*D*—H	H···*A*	*D*···*A*	*D*—H···*A*
C3—H3···O4^i^	0.95	2.54	3.480 (2)	172
C12—H12···O3^ii^	0.95	2.37	3.281 (3)	161
C38—H38···N2^iii^	0.95	2.60	3.462 (5)	151

Symmetry codes: (i) *x*+1, *y*, *z*; (ii) −*x*+2, −*y*, −*z*; (iii) *x*−1, *y*, *z*.

## References

[bb1] Bruker (2013). *SAINT*. Bruker AXS Inc., Madison, Wisconsin, USA.

[bb2] Bruker (2014). *APEX2* . Bruker AXS Inc., Madison, Wisconsin, USA.

[bb3] Dong, K., Fang, X., Gülak, S., Franke, R., Spannenberg, A., Neumann, H., Jackstell, R. & Beller, M. (2017). *Nat. Commun.* **8**, 14117–14124. 10.1038/ncomms14117 PMC5288498 28120947

[bb4] Eastham, G. R., Cameron, P. A., Tooze, R. P., Cavell, K. J., Edwards, P. G. & Coleman, D. L. (2004). World Patent. WO2004014552A1.

[bb5] Krause, L., Herbst-Irmer, R., Sheldrick, G. M. & Stalke, D. (2015). *J. Appl. Cryst.* **48**, 3–10. 10.1107/S1600576714022985 PMC4453166 26089746

[bb6] Sheldrick, G. M. (2008). *Acta Cryst.* A**64**, 112–122. 10.1107/S0108767307043930 18156677

[bb7] Sheldrick, G. M. (2015*a*). *Acta Cryst.* A**71**, 3–8.

[bb8] Sheldrick, G. M. (2015*b*). *Acta Cryst.* C**71**, 3–8.

[bb9] Westrip, S. P. (2010). *J. Appl. Cryst.* **43**, 920–925.

